# DeepFocus: Detection of out-of-focus regions in whole slide digital images using deep learning

**DOI:** 10.1371/journal.pone.0205387

**Published:** 2018-10-25

**Authors:** Caglar Senaras, M. Khalid Khan Niazi, Gerard Lozanski, Metin N. Gurcan

**Affiliations:** 1 Center for Biomedical Informatics, Wake Forest School of Medicine, Winston-Salem, NC, United States of America; 2 Department of Pathology, The Ohio State University Wexner Medical, Columbus, OH, United States of America; Taipei Medical University, TAIWAN

## Abstract

The development of whole slide scanners has revolutionized the field of digital pathology. Unfortunately, whole slide scanners often produce images with out-of-focus/blurry areas that limit the amount of tissue available for a pathologist to make accurate diagnosis/prognosis. Moreover, these artifacts hamper the performance of computerized image analysis systems. These areas are typically identified by visual inspection, which leads to a subjective evaluation causing high intra- and inter-observer variability. Moreover, this process is both tedious, and time-consuming. The aim of this study is to develop a deep learning based software called, DeepFocus, which can automatically detect and segment blurry areas in digital whole slide images to address these problems. DeepFocus is built on TensorFlow, an open source library that exploits data flow graphs for efficient numerical computation. DeepFocus was trained by using 16 different H&E and IHC-stained slides that were systematically scanned on nine different focal planes, generating 216,000 samples with varying amounts of blurriness. When trained and tested on two independent datasets, DeepFocus resulted in an average accuracy of 93.2% (± 9.6%), which is a 23.8% improvement over an existing method. DeepFocus has the potential to be integrated with whole slide scanners to automatically re-scan problematic areas, hence improving the overall image quality for pathologists and image analysis algorithms.

## Introduction

High-quality digital slides are becoming a ubiquitous and indispensable clinical workflow and research in pathology. Along with spatial resolution and color depth, image sharpness is often used to gauge the quality of digital slides. Most modern scanners come equipped with autofocus (AF) optics system to select focal planes to accurately capture three-dimensional tissue morphology as the best two-dimensional digital image. AF optics systems determine a set of focus points at different focal planes to be perfectly aligned with tissue height that may slightly vary within a slide. From these focal planes, scanners capture images to produce sharp tissue representation. However, commercial scanners may still produce digital images with out-of-focus/blurry areas if their AF optics system erroneously selects focus points that lie in a different plane than the proper height of the tissue [1]. The distance of the focus points from the actual tissue plane is proportional to the amount of blurriness, i.e. the larger the distance, the more blurriness it will result. When parts of an image are blurry, this affects the performance of both pathologists and automated image analysis algorithms. If these areas can be identified, the slides can be rescanned with additional focus points in areas of blurring. If the amount of blurring is minimal (acceptable levels are to be determined in advance), the resulting images can still be presented to pathologists or algorithms. Currently, blurry regions are identified manually, a process that is subjective, tedious, error-prone, and time-consuming [2]. It also disrupts the workflow as it will be easy to miss blurry regions until the time of clinical review, which will delay the diagnosis of the case.

In recent years, a few systems have been developed to identify scanning artifacts automatically from digital slides [1, 3–6]. Walkowski and Szymas [3] developed an algorithm to compare the quality of digital slides generated by different scanners. They scanned the same glass slide with multiple scanners and noticed that each scan resulted in a constant amount of translation in the planer space, which made it difficult to fairly compare among digital slides. The resulting digital slides were manually registered to compensate for the constant translation. To reduce the computational complexity, the algorithm randomly selected relatively small areas that correspond to the same fragments among digital images of the same slide. In these small areas, Gray Level Co-occurrence Matrix (GLCM) was computed, which aggregated the distribution of co-occurring values [7]. The authors also used the GLCM to compute contrast and entropy statistics, which were then used to compare the quality of images captured by different scanners. Although the authors did not explicitly propose an algorithm to identify blurry regions, the algorithm can easily be adapted to identify such regions.

In another study, Zerbe et al. developed a distributed image analysis system to calculate the amount of sharpness of image patches, classifying each patch into one of the four sharpness categories: excellent, okay, review and defective [5]. The sharpness was computed by a modified Tenenbaum gradient (Tenengrad) operator [8]. In a similar study, Hashimoto et al. proposed a method that is capable of assessing both blur and noise by an evaluation index [4]. The index was calculated by linear regression analysis using the sharpness and noise information from the training dataset. The training dataset was selected according to the intended purpose of the image quality evaluation, i.e. clinical usage or image analysis. The method was tested with both objective and subjective image quality measurements on small regions sampled from a single hematoxylin and eosin (H&E) digital slide. Because both training and test patches were extracted from the same H&E digital slide, it’s hard to assess how generalizable this method is.

Lahrmann et al. presented an algorithm to overcome the difficulties of the liquid-based cytology scanning [6]. The algorithm first performs a systematic analysis of the height variations within cytological samples in the z-dimension. Then, it performs a cell-based analysis to decide whether a focus point is valid, i.e. a cell, not an artifact, is detected. After the sample is imaged with the focus points, the algorithm divides the image into 16 sub-regions and then detects cells in each sub-region by their color intensity values. In the experiments, for each sub-region, 200 of the detected cells were classified as sharp or blurred by a Support Vector Machine classifier using five gradient based features. The percentage of in-focus cells (0–100%) defines a score for each region, and a combination of these scores in a slide determines its sharpness. If the image quality is below a threshold, the algorithm reselects other focus points and starts the sharpness calculation again.

Lopez et al. proposed another approach to detect blurred regions due to an incorrect (or suboptimal) focusing during acquisition [1]. To train their algorithm, they used 48,000 tiles of size 200x200 pixels at 20x magnification. For each tile, the algorithm extracted a set of features: Haralick features from GLCM and the Tenengrad operator. Using these features, a Decision Tree classifies each test tile as in-focus or blurred. To reduce the number of false positives, the algorithm applied a gray-scale morphological closing followed by a gray-scale morphological opening with a 3x3 structuring element. To validate the method, an expert randomly selected blurry and sharp tiles from digital slides of H&E and immunohistochemically (IHC) stained glass slides and evaluated the classification performance on these 3,438 tiles. A similar approach was proposed by Jimenez et al. which starts by extracting the tissue map using Otsu thresholding [2]. The algorithm divided the tissue into 64x64 pixel tiles. For each tile, the Cumulative Probability of Blur Detection contrast, entropy, and Tenengrad statistics were calculated. Each of these statistical measures was subjected to thresholding to decide if the tile under consideration is in focus or blurry. Ties were resolved by the majority voting algorithm [9]. To reduce classification errors, the resulting map was smoothed by application of morphological closing followed by a morphological opening with a disc-shaped structuring element.

**Fig 1:**
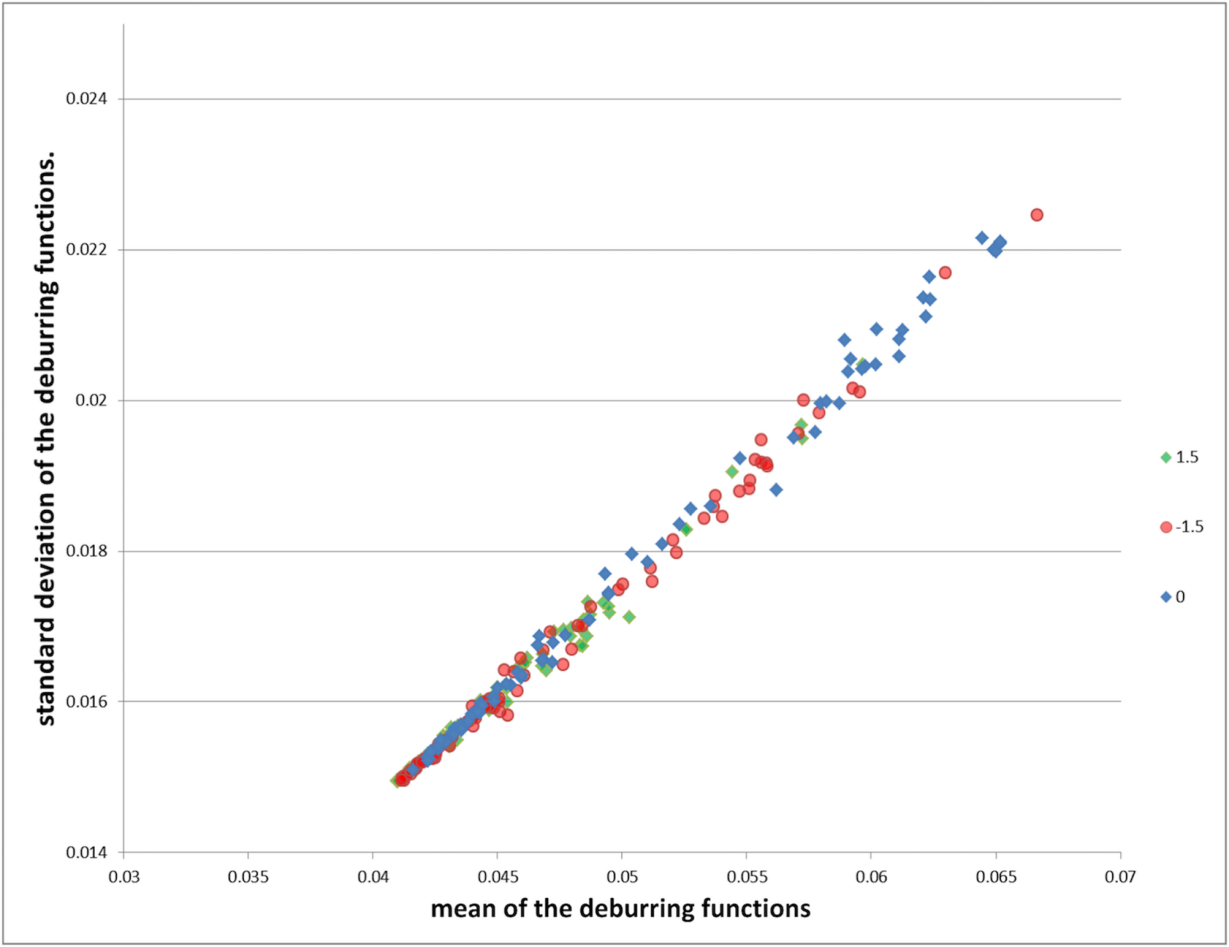
The mean and standard deviation of the resulting deburring functions for each tile. The tiles corresponding to infocus tiles are represented by blue dots. The blurred tiles obtained on −1.5 μm, 1.5 μm focal planes offsets are represented by red and greed dots, respectively.

Although all these studies have shown promising results, they were not systematically validated to justify how they generalize to unseen data, or how well they perform for varying amounts of blurriness. To illustrate the complexity of the blur detection problem, we randomly selected 80 tiles from four different slides (with different amount of blurring) and subjected them to a blind deconvolution process [10]. We initialized the blind deconvolution with a Gaussian function (zero mean and standard deviation two) and estimated the deblurring function via maximum likelihood. Fig 1 shows the mean and standard deviation of the resulting Gaussian deburring functions. From this figure, it is evident that a family of deburring functions would be necessary to recover the correct amount of blurring from histology specimens. Considering variation and the amount of non-linearity involved in the process, we have decided to develop a deep learning [11] based approach, which is a better choice than the conventional image analysis methods that were considered for this task [1, 3–6].

The success of deep learning over conventional image analysis methods [12, 13] is mainly attributed to its ability to identify discernable features without human intervention. Deep learning has recently been successfully applied in digital pathology to detect and segment nuclei and for diagnostic classification [14–17]. However, to the best of our knowledge, deep learning has not been yet used to identify blurry regions from digital pathology images.

In this paper, we propose a novel convolutional neural network to atuomatically identify out of focus regions in histopathological images. Our method is novel in terms of: 1) data curation, 2) generalization to different types of tissues, 3) being agnostic to H&E and IHC staining, 4) and in terms of algorithimic eficiency.

## Materials

### Training and validation datasets

**Fig 2:**
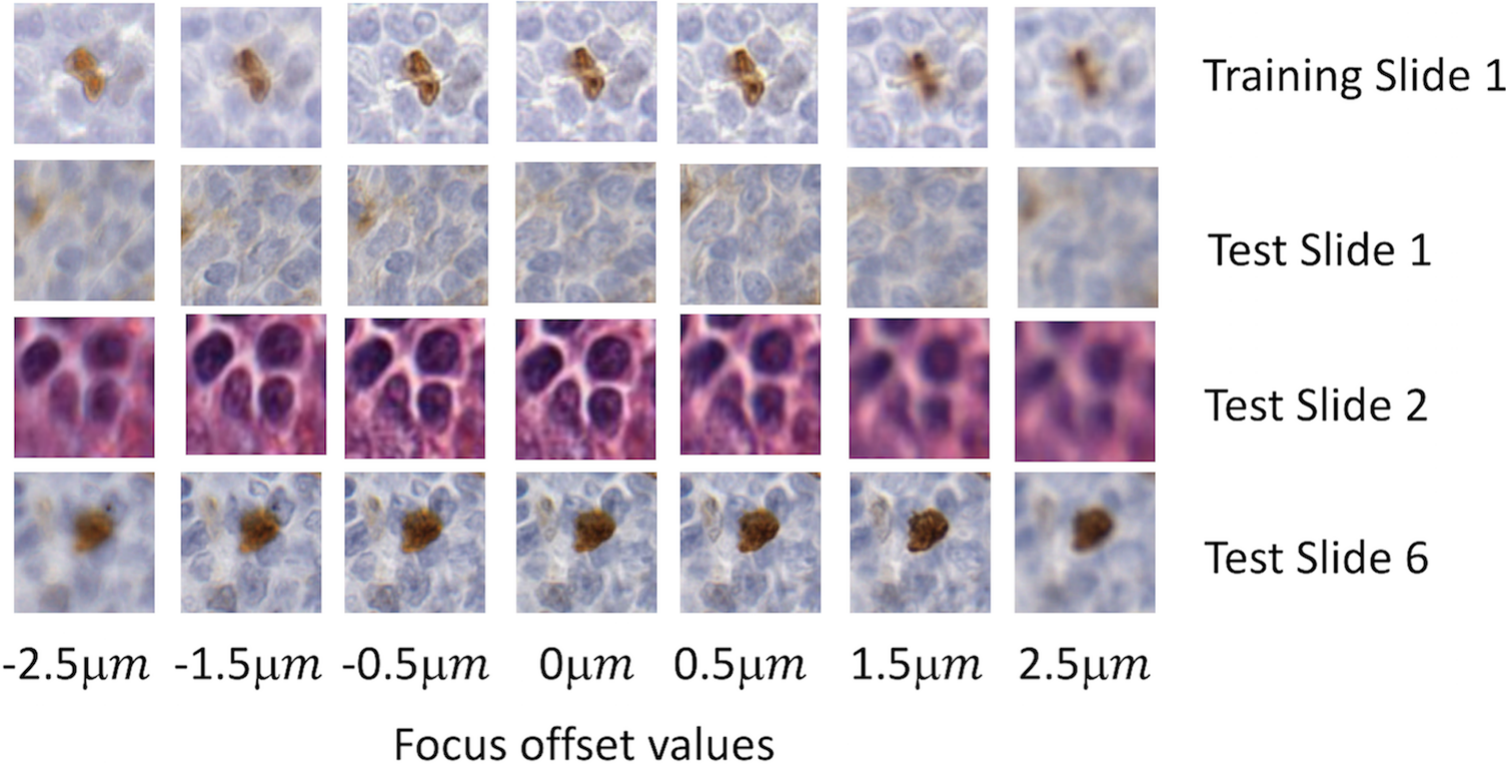
Example images cropped from digital slides with different focus offset values. The first row is generated from one of the training slides. The other two rows are generated from testing slides.

Our training dataset contained four digital slides with different stains (H&E, Ki67, CD21, and CD10) from four different patients, i.e. there were 16 slides. For each slide, we scanned a region of interest (ROI) of approximately 6 mm^2^ area, with Aperio ScanScope (Leica Biosystems Inc., Buffalo Grove, IL) at a 40x magnification where the pixel size is 0.2461 μm x 0.2461 μm. For each ROI, a trained operator manually selected 25 focus points and fine-tuned the autofocus values of the selected points to ensure that the focal planes align well with the tissue height. Then, the operator perturbed the focus points with a fixed offset value, Ω, where Ω ∈ {−2.5 μ*m*, −2.0 μ*m*, −1.5 μ*m*, −0.5 μ*m*, 0.5 μ*m*, 1.5 μ*m*, 2 μ*m*, 2.5 μ*m*} (Fig 2). This method enabled us to obtain the same ROI with different focal planes, some of which not aligning with the proper tissue height, resulting in different levels of blurring. Finally, all of these ROI images were divided into 64x64 pixels size tiles and 2500 of these tiles were randomly selected, resulting in a total of 360,000 tiles. In order to create ground truth, the tiles whose offset values between [-0.5 μm, 0.5 μm] are labeled as in-focus and the rest of the images were labeled as blurry. The in-focus range (i.e. [-0.5 μm, 0.5 μm]) was empirically determined as it was practically impossible for an expert observer to differentiate between these ROIs visually, and inter- and intra-reader variability could play a role. Since the number of blurry tiles (240,000) was larger than the number of in-focus tiles (120,000), 108,000 sample tiles sampled from four different stains, were randomly selected from each class for training to prevent training set imbalance in categories. Lastly, ten percent of the sample tiles (i.e. 21,600) were selected as the validation set.

This study is IRB approved by the Ohio State University (Study Number: 2007C0069), Cancer Institutional Review Board, with Waiver of Consent Process, and Full of Waiver of HIPAA Research Authorization. Furthermore, all samples were fully anonymized by the rules set by the Ohio State University, Cancer Institutional Review Board.

### Testing dataset

For testing, we used two different datasets, the first of which was generated by the same scanner that produced our training data. We acquired six additional digital slides (3 H&E, 2 Ki67, and 1 CD10 cases) to form the first test dataset. Fig 2 shows some of the example images cropped from these digital slides with different focus offset values. The digital slides resulted in a total of 218,304 in-focus and 436,608 blurred tiles. To compare our results with those in presented in Lopez et al.’s work [1], we down-sampled the images to 20x magnification and obtained 200x200 tiles (6,168 in-focus tiles and 12,336 blurred tiles). Importantly, the tile size used in [1] is 36 times coarser than that of our approach.

The second test dataset, which was acquired with a Hamamatsu NanoZoomer 2.0HT scanner (Hamamatsu, Japan), consists of two H&E slides. Because these images were acquired at another facility, the amount of blurring was completely random; therefore, we visually evaluated the performance of the proposed method on these slides for comparison.

## The deep learning architecture

**Fig 3:**
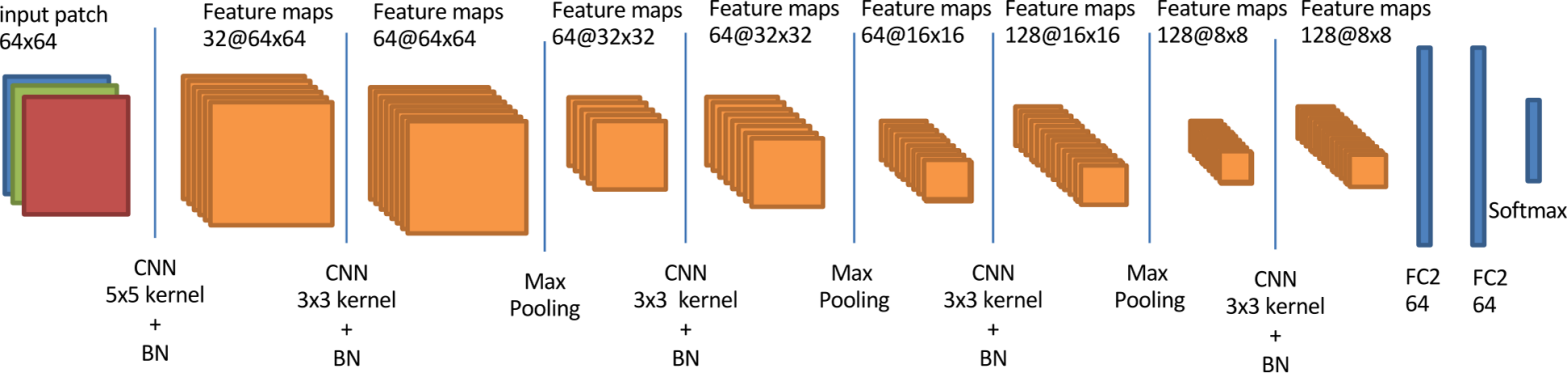
Architecture of the DeepFocus.

In this study, we designed a convolutional neural network based system called DeepFocus, to classify each image tile as either in-focus or blurry. DeepFocus consists of five convolution layers, three max-pooling layers (after the third, fourth and fifth convolution layers) and fully connected layers (Fig 3). The last layer in DeepFocus is a softmax, which results in a probability of a tile belonging to either of the two classes. We defined the objective function as categorical cross entropy between the label and the prediction.

### Preprocessing

Data preprocessing plays a critical role in many deep learning algorithms [18]. Several studies have reported better results when data is scaled between zero and one [19]. For this reason, we linearly scaled the color intensities between 0 and 1. We further applied zero-centering to transform the data in such a manner that all images in the dataset have the same average value of zero.

## Data augmentation

With the exception of transfer learning, deep networks often require a huge number of training samples to achieve satisfactory classification accuracy. Because of the limited number of images in our training dataset, we employed commonly used data augmentation approaches that increase the number of samples by randomly 1) flipping the image in the vertical and horizontal direction and 2) rotating the image by 90^0^.

### Design details and parameter optimization

During the training, we used Stochastic gradient descent (SGD) [20] and mini-batches consisting of 64 tiles. The operation performed by a layer, **x**, can be represented as **x** *= g(*W***u****+b)* where *W (weights)* and *b(bias)* are the parameters to be learned, *g* is an activation function, and **u** is the input vector from the previous layer. At each layer, we used Rectifier Linear Unit (ReLU), g = max(0,Wu + b), as the activation function as its simplistic nature coupled with SGD results in faster learning [21].

Like other optimization algorithms, SGD requires initial values for model parameters and each layer’s input is affected by the parameters of the previous layers [22]. As a result, a small change in the previous layer’s parameters is amplified as the network becomes deeper. The inconsistency in the distribution of layers’ inputs causes a problem, called internal covariate shift, since the layers need to adapt to the new distribution continuously. To mitigate the effects of this problem, we used Batch Normalization (BN) [22]. For a given mini-batch, the input vector of a layer was represented by u. We calculated the mean (${\bar{u}}_{i}$) and variance ($\sigma_{i}^{2}$) of each feature, *i*, at each layer. Subsequently, we normalized *u*_*i*_ as follows

$${\hat{u}}_{i}=\frac{u_{i}-{\bar{u}}_{i}}{\sqrt{\sigma_{i}^{2}+\epsilon }},$$ (1)

where ϵ is a small constant (ϵ = e^-15^). To increase the representational power, we scaled and shifted the normalized value by using additional tunable parameters γ and β:

$${BN}_{\gamma ,\beta }(u_{i})=\gamma \;{\hat{u}}_{i}+\beta ,$$ (2)

Although Ioffe used the BN before RELU in [22], there is growing evidence that BN results in quicker convergence if applied after ReLU [18]; therefore, we opted for BN after ReLU. To reduce the risk of overfitting, we used Dropout regularization in the fully connected layers [23]. Training with BN is known to have a regularization effect, so we reduced the dropout strength to p = 0.2 [23]. We implemented DeepFocus using TensorFlow [24].

The training phase, ran on the Owens supercomputer at Ohio Supercomputer Center (OSC) (Tesla P100-PCIE-16GB), took about 180 seconds per epoch. From the training, we realized that the underlying function is relatively smooth as SGD was able to achieve relatively high classification accuracy in a few iterations. We stopped the training at the end of 20^th^ epoch since the validation accuracy stopped increasing. The hyper-parameters of the architecture (kernel size, the number of layers and learning rates) were tuned using grid search and cross-validation on the validation [25]. Table 1 shows the explored and selected values for our hyper-parameters.

**Table 1:** Parameter values explored for each hyperparameter. The bold ones represent the selected parameter using grid search [25].

**Hyperparameter**	**Optimization Space**
CNN—Layer 1 Kernel size	[3*x*3,**5*x*5**,7*x*7,9*x*9]
CNN—Layer 2 Kernel size	[**3*x*3**,5*x*5,7*x*7]
Learning rate	[**0.01**,0.06,0.12,0.22]
Batch size	[32,**64**,128]

## Evaluation methodology

**Fig 4:**
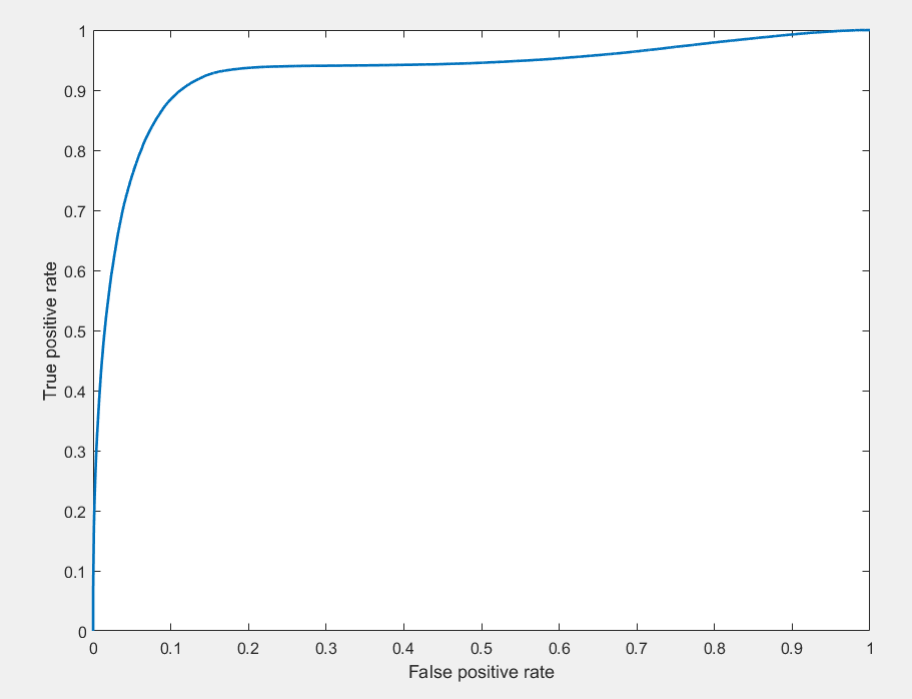
The ROC curve for the proposed approach.

To evaluate the proposed method, we considered a tile as in-focus if the probability of being in-focus is higher than 0.5, then we measured the accuracy:

$$Accuracy=\frac{TP+TN}{P+N}$$ (3)

where TP, TN, P, and N correspond to the numbers of correctly classified in-focus tiles, the number of correctly classified blurry tiles, the total number of in-focus tiles and total number blurry tiles, respectively. Additionally, the Receiver Operating Characteristic (ROC) curve is plotted to observe TP and TN rates (Fig 4).

The mean, $\bar{A_{\Omega }}$, and standard deviation, *σ*(*A*_*Ω*_), accuracy for a specific focus offset values were calculated where ***A***_**Ω**_^***i***^ represents the accuracy of the algorithm on image i, with the specific focus offset value, Ω.

## Results and discussion

### Comparison with a prior method for different offset values

We compared our proposed method, with state of the art algorithm, proposed by Lopez et. al. [1] Since the Lopez algorithm is designed to analyze 200x200 images captured at 20X magnification, we downsampled the test images to accommodate for these differences. Table 2 shows the average accuracy values of the two different approaches for different focus offset values. The fourth column shows the difference in accuracy between the two approaches. On average, DeepFocus is 23.8% more accurate than Lopez’s approach [1] and the variabilty (as measured by ***σ***) is less. In our previous studies, we did extensive analysis of optimization approaches and how they can help with achieve better accuracies [26, 27]. Considering that our current performance is 93.2% and the state of the art performance is 69.4%, the current optimization seems to be satisfactory.

**Table 2:** The comparison of the proposed approach (DeepFocus) and Lopez's approach [1] in terms of accuracy on the first test dataset.

	$\bar{A_{\Omega }}$ **(Eqn. 4)**			***σ*(*A***_**Ω**_**) (Eqn. 5)**	
Ω (μ*m*)	DeepFocus	[1]	|DeepFocus—[1]|	DeepFocus	[1]
**-2.5**	99.2%	78.5%	20.3%	1.7%	39.0%
**-2**	90.1%	56.8%	33.6%	18.0%	41.2%
**-1.5**	69.7%	35.9%	31.1%	35.7%	39.5%
**-0.5**	99.1%	78.1%	20.4%	0.6%	38.8%
**0**	96.9%	79.0%	17.5%	5.0%	39.0%
**0.5**	90.6%	78.9%	11.1%	15.5%	39.3%
**1.5**	93.2%	45.4%	47.8%	12.7%	44.8%
**2**	99.9%	74.6%	24.9%	0.3%	36.0%
**2.5**	100.0%	97.2%	2.8%	0.1%	5.6%
**Average**	93.2%	69.4%	23.8%	16.6%	39.2%
**Std**	9.6%	19.4%			

### Slide-based comparison

**Fig 5:**
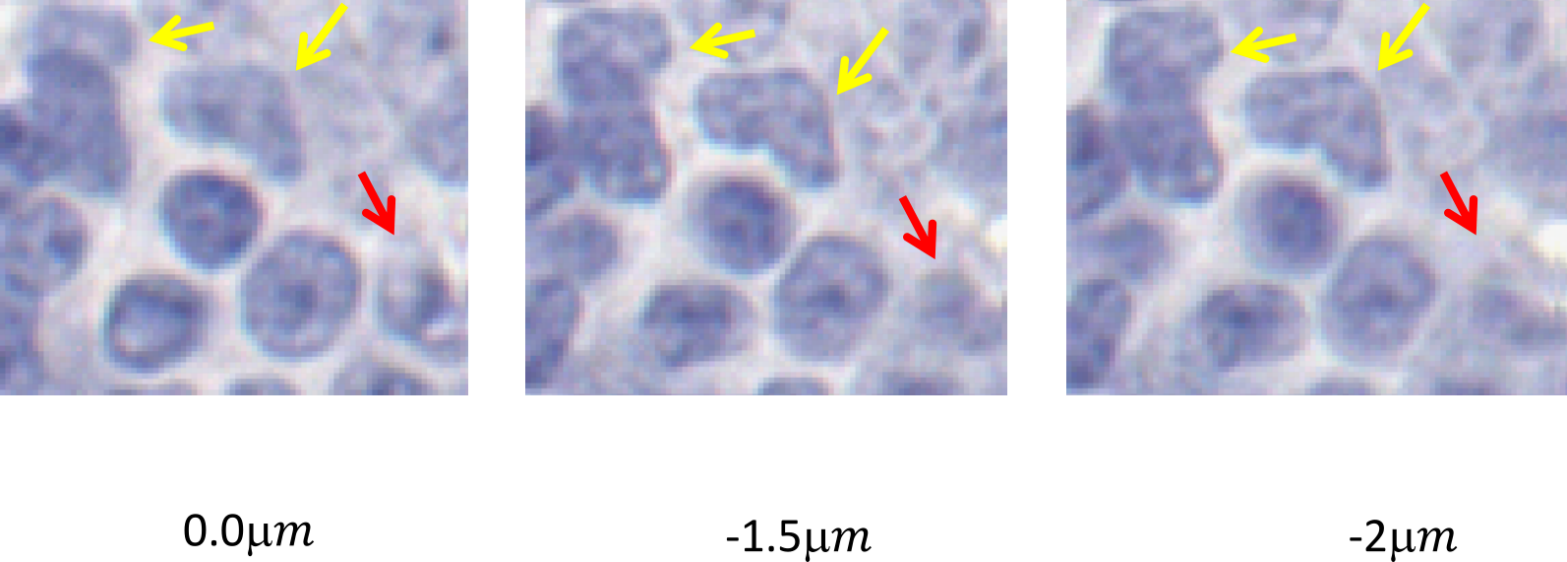
An example case where some of the nuclei look sharper in different focal levels (Slide 4). Yellow arrows show some of the in focused nuclei in negative offset values. The positive arrow shows some of the focused nuclei in zero offset.

Table 3 compares of the proposed approach (DeepFocus) and Lopez's approach [3] for each slide on the test dataset. This experiment revealed that DeepFocus performs better than the Lopez's approach [1] for all offset values between -2.5 and 2.5. We also observed that Lopez's approach struggled in identifying moderately blurry regions (i.e. 0.5 μ*m* < |Ω| < 2 μ*m*). It is worth mentioning that the standard deviation of DeepFocus for the offset values in the range [−2 μ*m and* −1.5μ*m*] is higher than the other offset values. Due to the finite thickness of the slides (5 μ*m*), digital images represent nuclei in different focal planes. Therefore, with negative offset values, the scanner focused on some of the nuclei and created a sharper image for these regions. The Slide 1 and Slide 4 demonstrate typical examples for this type of problem (Fig 5). Interestingly, Lopez’s approach failed to identify in-focus regions in Slide 1.

**Table 3:** The comparison of the proposed approach (DeepFocus) and Lopez's approach [3] for each slide on the test dataset.

	**Slide 1** **(CD 10)**		**Slide 2** **(H&E)**		**Slide 3** **(H&E)**		**Slide 4** **(Ki67)**		**Slide 5** **(H&E)**		**Slide 6** **(Ki67)**	
Ω (μ*m*)	Deep Focus	Lopez et. Al.	Deep Focus	Lopez et. Al.	Deep Focus	Lopez et. Al.	Deep Focus	Lopez et. Al.	Deep Focus	Lopez et. Al.	Deep Focus	Lopez et. Al.
**-2.5**	99.8%	100.0%	100.0%	73.1%	100.0%	1.9%	95.8%	99.2%	100.0%	100.0%	99.7%	97.0%
**-2**	88.0%	100.0%	100.0%	10.8%	100.0%	0.3%	54.6%	76.1%	100.0%	68.6%	98.3%	84.9%
**-1.5**	36.5%	100.0%	99.5%	7.8%	96.5%	0.0%	17.2%	38.6%	99.7%	6.0%	68.7%	62.8%
**-0.5**	98.5%	0.0%	99.8%	99.7%	98.6%	100.0%	99.5%	85.6%	99.7%	87.3%	98.8%	96.1%
**0**	87.2%	0.0%	96.2%	99.2%	99.5%	100.0%	98.9%	86.9%	99.9%	92.1%	99.8%	96.1%
**0.5**	59.6%	0.0%	99.6%	100.0%	98.2%	100.0%	91.0%	82.2%	98.1%	99.7%	97.4%	91.8%
**1.5**	99.5%	100.0%	100.0%	5.6%	100.0%	0.0%	91.6%	98.6%	100.0%	21.9%	68.3%	46.4%
**2**	99.9%	100.0%	100.0%	64.7%	100.0%	6.9%	99.9%	100.0%	100.0%	96.5%	99.3%	79.3%
**2.5**	100.0%	100.0%	100.0%	100.0%	100.0%	86.1%	100.0%	100.0%	100.0%	100.0%	99.7%	97.0%
**Slide Avg**	85.4%	66.7%	99.5%	62.3%	99.2%	43.9%	83.2%	85.2%	99.7%	74.6%	92.2%	83.5%
**Std.**	22.6%	50.0%	1.2%	42.7%	1.2%	50.1%	28.6%	19.6%	0.6%	36.0%	13.5%	17.9%

### Evaluation on a different scanner

**Fig 6:**
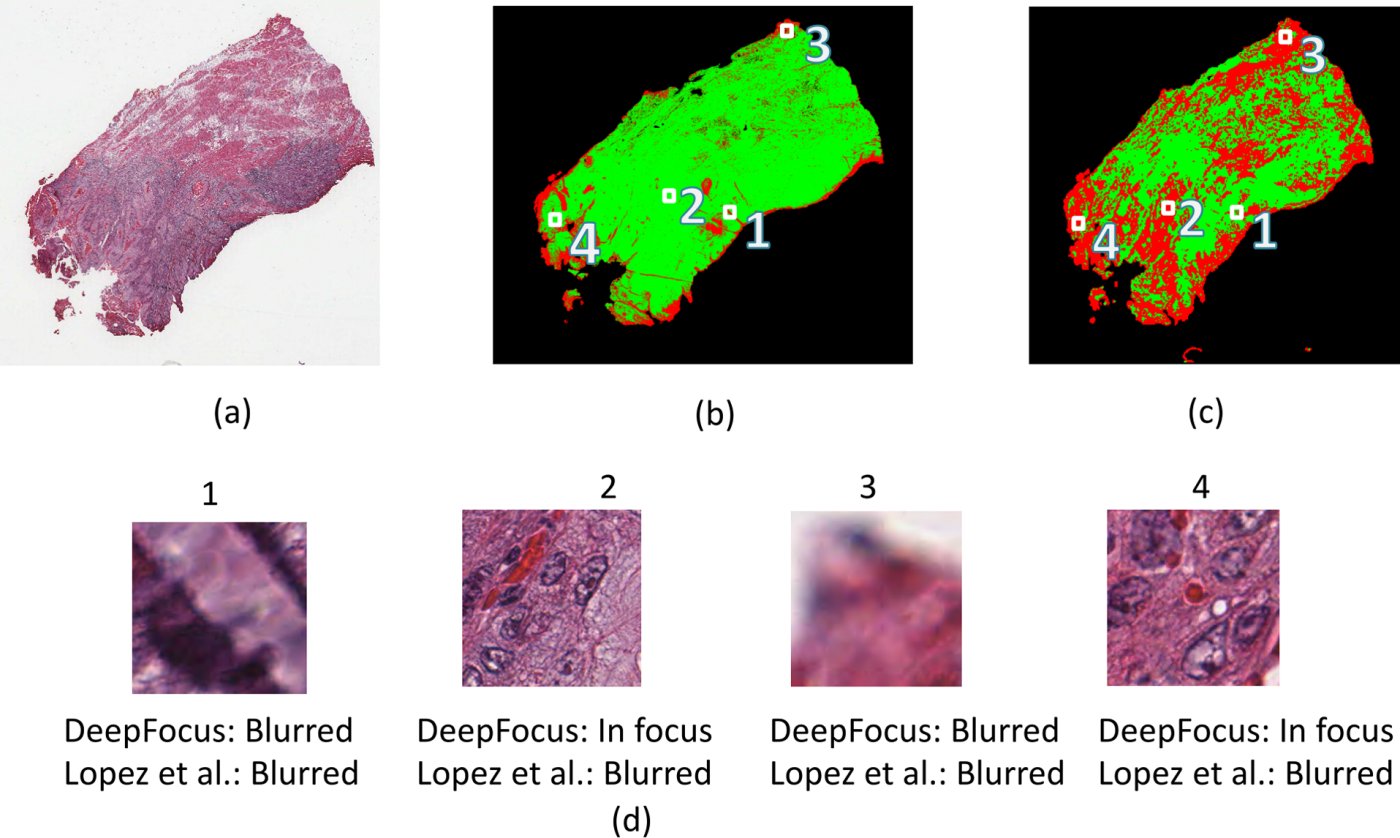
The outputs of the DeepFocus (b) and Lopez’s approach(c) on a H&E slide (a) acquired with a different scanner where the green and red colors represent in-focus and blurry regions, respectively.

We also evaluated the robustness of the DeepFocus on full digital slides in the second test dataset that was acquired with a different scanner. The images were approximately of size 95,000 x 70,000 pixels. To avoid analyzing the tiles outside the tissue area, we find the tissue map at 1x magnification of the entire slide by Otsu Thresholding [28]. Once the tissue is detected, we classified each non-overlapping tile (64x64 pixels) using DeepFocus to create a binary mask of the same size an input digital slide. Fig 6B, shows an example mask for one of the whole slide images (Fig 6A) where the green and red colors represent in-focus and blurry regions, respectively. As proposed in [1], this mask can be used to generate new focus points for the scanner, resulting in a higher quality image. Fig 6C shows the output of the Lopez’s approach for the same image. Fig 6D shows some of the in-focus and blurred regions identified by DeepFocus and how these areas are labeled by the Lopez’s algorithm.

### Functional comparision with existing methods

In this study, we designed a new convolutional neural network, called *DeepFocus*, to identify blurry regions. Unlike prior studies, we systematically acquired data at different focal planes to vary the amount of blurring in a controlled manner, and performed rigorous validation on independent test sets. While existing algorithms can take a long time to run, we managed to achieve reasonable execution times (around 10 minutes) for a full digital slide (16GB) at 40x magnification. We also demonstrated that our method generalizes to multiple stain types (i.e. both H&E and IHC). Lastly, we compared the proposed method with a recently published study [1] in terms of accuracy, robustness and computational complexity. Unlike DeepFocus, the other apporoach was designed to work at 20x magnification and the analyzed unit area was 36 times larger than our’s, resulting in a coarser output. A functional comparison between DeepFocus and the previous methods is summarized in Table 4.

**Table 4:** Functional comparison of the proposed method with previous studies.

Approach	Purpose	Graphical Processing Unit (GPU) support	Analyzed Unit Area (magnification)	Code availability	Tested on different stains	Tested on different local planes
Walkowski and Szymas [3]	Image quality comparison different devices	No	unknown	No	No	No
Hashimoto et al. [4]	out of focus	No	98μm x98μm (40x)	No	No	No
Zerbe et al. [5]	out of focus	No	unknown	No	No	No
Lahrmann et al. [6]	out of focus	No	unknown (20X)	No	Yes	No
Lopez et al. [1]	out of focus	No	98μmx98μm (20x)	Yes	Yes	No
DeepFocus (proposed)	out of focus	Yes	17μmx17μm (40x)	Yes	Yes	Yes

### Computational performance

We evaluated the speed performance on three different configurations. The first configuration is a workstation (XEON E3-1220 v5) without any GPU; the second is a laptop with Nvidia GTX 1050 GPU card, and the third configuration is Owens at Ohio Supercomputer Center (Table 5). The elapsed time includes reading the whole image, detecting the tissue using Otsu thresholding [28], and classifying 489,904 64x64 non-overlapping tiles belonging to the tissue with the pre-trained network. The algorithm took 643 seconds to analyze the whole data (at 40X magnification) on the laptop by using the GPU. In comparison, Lopez’s CPU-based approach took 713 seconds to analyze at 20X magnification, and their spatial result was 36 times coarser than that of DeepFocus.

**Table 5:** Elapsed Time on a whole slide image with 40X magnification (95,964x 69,315 pixel, 19 GB raw image).

Configuration	Implementation	Elapsed time
Workstation Computer	CPU (XEON E3-1220 v5, 64 GB Ram)	9,163 seconds
Laptop	GPU (GTX 1050- 4GB)	643 seconds
Owens (OSC) [29]	GPU(Tesla P100-PCIE-16GB)	441 seconds

## Conclusions

In this study, we proposed a novel deep learning framework, DeepFocus, to identify blurry regions in digital slides. The novelty of the study lies in both the design of our DeepFocus framework as well as in its systematic evaluation. For training and testing, we carefully determined a set of focus points at different focal planes that conform well to the tissue morphology. These points were perturbed in a systematic manner to produce different amount of blurring in digital slides. The robustness of DeepFocus to disease and stain variations was validated by 1) creating independent training and test datasets with digital images from patients with different diseases, 2) acquiring images from different scanners and laboratories. Comparision of DeepFocus with an existing method [1], resulted in 23.8% higher accuracy on average with smaller variation.

The algorithm can be used in conjunction with scanners and image analysis algorithms to identify out-of-focus regions. Likewise, a pathologist can use it to automatically exclude out-of-focus regions from further analysis. To make our proposed method responsive to the needs of different kinds of users (e.g. engineers, image analysts, or pathologists), it needs to have a low computational overhead. To improve computational efficiency to the proposed method, we opted for tile sizes of 64x64 pixels. This tile size provides a good tradeoff between computational efficiency and granularity/accuracy of identifying out-of-focus regions. If a higher level of accuracy is required, we can opt for either overlapping tiles or relatively small tiles but at the cost of higher computational overhead.

Like most of the deep learning approaches, DeepFocus involves matrix multiplication and convolution, which can be parallelized. Since GPU has a massively parallel architecture compared to CPU, it is advantageous for this task. DeepFocus benefits from the technological improvements in GPU which enables it to analyze a digital slide in a matter of a few minutes on a standard laptop computer. Recent studies show that the GPU based deep learning approaches speed up by 50x in just three years and researchers are expecting another 10x boost in the next few years [30]. As a result, we expect DeepFocus to become much faster in the near future.

Although deep learning techniques are being successfully applied to other digital pathology problems, DeepFocus is the first implementation of such techniques to characterize problems of digital image generation in pathology. This first application focused on the problem of accurately identifying blurry (out-of-focus) regions in whole slides images. These problems are very common as Stathonikos, et al. document, 5% of the cases had problems with scanning artifacts, such as blurry images and incomplete slides in the Dutch digital pathology experience [31]. Digital imaging problems are not limited to blurring; tissue folding, over- or under-staining, air-bubbles, compression artifacts are some of the many other problems. The DeepFocus framework needs to be extended to identify these problems before the images reach pathologists or image analysis algorithms. In future, we are planning on expanding our dataset to include more disease categories, different types of stains as well as other types of scanners. Additionally, we are planning to estimate the focal offset error which may be useful during the rescanning.

### Code availability

The source code for running DeepFocus on a whole slide is available from https://github.com/cialab/DeepFocus
